# Neighborhood clustering of non-communicable diseases: results from a community-based study in Northern Tanzania

**DOI:** 10.1186/s12889-016-2912-5

**Published:** 2016-03-05

**Authors:** John W. Stanifer, Joseph R Egger, Elizabeth L. Turner, Nathan Thielman, Uptal D. Patel, for the Comprehensive Kidney Disease Assessment for Risk factors, epidemiology, Knowledge, and Attitudes (CKD AFRiKA) Study

**Affiliations:** Department of Medicine, Duke University, DUMC Box 3182, Durham, NC 27710 USA; Duke Global Health Institute, Duke University, Durham, NC 27710 USA; Duke Clinical Research Institute, Duke University, DUMC Box 3646, Durham, NC 27710 USA; Department of Biostatistics and Bioinformatics, Duke University, DUMC Box 2721, Durham, NC 27710 USA; Duke University Medical Center, Box 3182, Durham, NC 27710 USA

**Keywords:** Chronic kidney disease, Cluster design, Design effect, Epidemiology, Intra-cluster correlation, Non-communicable disease, Sub-Saharan Africa, Variance

## Abstract

**Background:**

In order to begin to address the burden of non-communicable diseases (NCDs) in sub-Saharan Africa, high quality community-based epidemiological studies from the region are urgently needed. Cluster-designed sampling methods may be most efficient, but designing such studies requires assumptions about the clustering of the outcomes of interest. Currently, few studies from Sub-Saharan Africa have been published that describe the clustering of NCDs. Therefore, we report the neighborhood clustering of several NCDs from a community-based study in Northern Tanzania.

**Methods:**

We conducted a cluster-designed cross-sectional household survey between January and June 2014. We used a three-stage cluster probability sampling method to select thirty-seven sampling areas from twenty-nine neighborhood clusters, stratified by urban and rural. Households were then randomly selected from each of the sampling areas, and eligible participants were tested for chronic kidney disease (CKD), glucose impairment including diabetes, hypertension, and obesity as part of the CKD-AFRiKA study. We used linear mixed models to explore clustering across each of the samplings units, and we estimated absolute-agreement intra-cluster correlation (ICC) coefficients (*ρ*) for the neighborhood clusters.

**Results:**

We enrolled 481 participants from 346 urban and rural households. Neighborhood cluster sizes ranged from 6 to 49 participants (median: 13.0; 25th–75th percentiles: 9–21). Clustering varied across neighborhoods and differed by urban or rural setting. Among NCDs, hypertension (*ρ* = 0.075) exhibited the strongest clustering within neighborhoods followed by CKD (*ρ* = 0.440), obesity (*ρ* = 0.040), and glucose impairment (*ρ* = 0.039).

**Conclusion:**

The neighborhood clustering was substantial enough to contribute to a design effect for NCD outcomes including hypertension, CKD, obesity, and glucose impairment, and it may also highlight NCD risk factors that vary by setting. These results may help inform the design of future community-based studies or randomized controlled trials examining NCDs in the region particularly those that use cluster-sampling methods.

## Background

Non-communicable diseases (NCDs) are a growing global epidemic that disproportionately affect low- and middle-income countries (LMICs) [1]. In sub-Saharan Africa, they are now one of the leading causes of death among adults, and in order to begin to address this burden, high quality community-based epidemiological studies from the region are urgently needed [2–5]. Additionally, outcomes-related research either through observational cohort studies or randomized-controlled trials (RCTs) will be an important component of the public health response moving forward [6].

Nonetheless, many challenges exist in carrying out these studies. Poor infrastructure and a lack of resources in many of the sub-Saharan African countries limit rigorous studies, in part due to inadequate methodological capabilities. Physical addresses, phonebooks, and reliable census data are often unavailable for many populations in the region which means that representative community-based samples often require labor-intensive, prospective household surveys. In this context, cluster-designed sampling methods offer an efficient, practical, and cost-effective means of obtaining a representative sample from the population of interest [7, 8].

However, studies that use cluster sampling methods require extra considerations in their design and analyses, and cluster-designed studies in sub-Saharan Africa continue to inadequately address many of these considerations [9]. Because study participants or households are drawn from clusters, which serve as the primary sampling unit, they can demonstrate more homogeneity than would otherwise be expected from a simple, random sample. For NCDs, similar lifestyles, environmental risks, economic stress, and genetic backgrounds may all increase homogeneity within clusters, and consequently, this increased homogeneity within clusters, or intra-cluster correlation (ICC), can significantly affect the precision of population parameter estimates [10, 11]. The ICC is typically quantified by the ICC coefficient, and although the ICC coefficient can be calculated post hoc during the analysis stage, this method may not be preferable or ethical in many sub-Saharan African settings due to cost and limited resources. Accounting for the design effect beforehand allows for more accurate estimations of sample size, budget requirements, and logistical needs; however, for NCD-related research, few ICCs have been reported in the region [9, 10].

The Comprehensive Kidney Disease Assessment for Risk Factors, epidemiology, Knowledge, and Attitudes (CKD-AFRiKA) study is an ongoing project in northern Tanzania with the goal of understanding and addressing the health burden of chronic kidney disease (CKD) and CKD-related NCDs. As part of the study, we conducted a cluster-designed, community-based epidemiologic survey. In the design stage, we were unable to identify any comparable ICCs for health outcomes related to CKD or CKD-related NCDs, and we had to extrapolate them from data derived from high-income settings. To fill this gap, we report here the observed intra-cluster correlations for multiple NCD-related factors from a community-based, sub-Saharan African setting [12].

## Methods

### Ethics, consent, and permissions

The study protocol was approved by Duke University Institutional Review Board (#Pro00040784), the Kilimanjaro Christian Medical College Ethics Committee (EC#502), and the National Institute for Medical Research in Tanzania. Written informed consent (by signature or thumbprint) was obtained from all participants.

### Study setting

We conducted a stratified, cluster-designed cross-sectional household survey between January and June 2014 in the Kilimanjaro Region of Tanzania, which has an adult population of more than 900,000 people [13, 14]. The region comprises seven districts, and our study was conducted in two of these districts, Moshi Urban and Moshi Rural, which served as strata for our sampling scheme. Within these districts, there are 21 and 31 administrative wards respectively that range in size from 1500 to 25,000 people. Each ward is then further sub-divided into neighborhoods (also known as streets). Neighborhoods are the most basic governmental administrative unit in Tanzania, and they range in population size from 500 to 5000 people. The 65 urban neighborhoods have a median population size of 2000 people and a median area of 0.50 km^2^. The 165 rural neighborhoods have a median population size of 2200 people and a median area of 4.00 km^2^. In total, there are 230 neighborhoods/streets in the Moshi Urban and Moshi Rural districts [14].

### Sampling methods

We used a three-stage cluster probability sampling method stratified by urban and rural. We used a random-number generator to select twenty nine neighborhoods within the Moshi Urban and Moshi Rural districts. We based the random neighborhood selection on probability proportional to size sampling according to the 2012 national census [14]. From the twenty-nine neighborhoods, we then randomly selected the starting point for each sampling area (37 in total) using geographic coordinates randomly generated by Arc Global Information Systems (ArcGIS), v10.2.2 (Environmental Systems Research Institute, Redlands, CA). From the randomly-selected geographic point, we then chose households based on a coin-flip and die-rolling technique (Appendix 1). All non-pregnant adults (age ≥ 18 years old) living in the selected households were recruited. A neighborhood cluster, therefore, included a group of individuals living in geographically-related households within the boundaries of an administrative neighborhood.

We targeted an enrollment between 15 and 25 participants per sampling area based on the requirements of the CKD AFRiKA study. The total sample size was designed to estimate the community prevalence of CKD with a precision of 5 % when accounting for the cluster-design effect, assuming a CKD prevalence up to 20 % and an ICC coefficient of 0.05. To reduce non-response rates, we attempted a minimum of two additional visits during off-hours (evenings and weekends) and multiple phone calls using mobile phone numbers.

### Data collection

Our data collection methods have been previously described in detail [12]. In brief, participants were tested for CKD and CKD-related conditions including diabetes and hypertension, and anthropomorphic data (including height, weight, and body mass index) were recorded for each participant.

CKD was defined as the presence of albuminuria (≥30 mg/dL; confirmed by repeat assessment) and/or a reduction in the estimated glomerular filtration rate (eGFR) ≤60 ml/min/1.73 m^2^ according to the Modification of Diet in Renal Disease equation without the race factor [15]. Hypertension was defined as a single blood pressure measurement of greater than 160/100 mmHg, a two-time average measurement of greater than 140/90 mmHg, or the ongoing use of anti-hypertensive medications. Glucose impairment was defined as an HbA1C >6.0 % in the presence or absence of ongoing treatment with anti-hyperglycemic medications. Diabetes mellitus was defined as an HbA1c level was ≥7.0 % or current known use of anti-hyperglycemic medications for the purpose of treating diabetes. Participants with an HbA1C between 6.0 % and 6.0 % in the absence of treatment with anti-hyperglycemic medications were considered to have pre-diabetes. Overweight was defined as a body mass index (BMI) greater than 25 kg/m^2^ and obesity was defined as a BMI greater than 30 kg/m^2^.

### Data analysis

We used STATA version 13 (STATA Corp., College Station, TX) for all data analyses. Continuous variables were summarized by the mean and standard deviation (SD) or median and inter-quartile range (IQR). Categorical variables were summarized using counts and percentages. To address potential non-response bias, mean and prevalence estimates were sample-adjusted using age- and gender-weights based on the 2012 urban and rural district-level census data [14]. To estimate the level of clustering in health outcome variables at the household level, the sampling area level, and the neighborhood cluster level, we first fitted a mixed effect model with separate random intercepts for neighborhood, sampling area, and household for each of the outcomes of interest. In these models, after accounting for neighborhood, very little clustering (<15 %) remained at the sampling area level and household level indicating that most of the variation in these outcomes was explained at the individual and neighborhood cluster-levels. As such, we estimated the ICC for the neighborhood clusters only.

To estimate the absolute-agreement ICC coefficient for neighborhood clusters (*ρ)* we used a one-way, random effects analysis of variance (ANOVA) estimator which has been shown to perform well for both binary and continuous outcomes across a wide range of *ρ* and cluster sizes [16–19]. These estimations were performed in STATA using the ‘loneway’ command which uses the F statistic to calculate *ρ* as described by Hayes and Moulton. Although alternative estimators are available for binary outcomes, given that the ANOVA estimator has been shown to perform well for binary outcomes, we chose to present all estimates based on the common, easily implementable approach as described above [17, 18].

We calculated *ρ* for the social characteristics, self-reported medical histories, physical and laboratory measurements, and measured health outcomes. Negative values were truncated at zero, and our reporting of *ρ* is in accordance with the guidelines suggested by Campbell et al. [20].

Variance estimation was based on asymptotic theory, as implemented in the ‘loneway’ command, which accommodates differing cluster sizes. The 95 % confidence intervals for each ICC coefficient were derived from the asymptotic standard error, which has been shown to provide good coverage probabilities for a wide range of parameter combinations including clusters, cluster sizes, and *ρ* [18, 21, 22]. Confidence intervals with negative values were truncated at zero.

## Results

Between January 2014 and June 2014, we enrolled 481 participants from 346 households from a total of 37 sampling areas (30 urban and 7 rural) within 29 neighborhoods (23 Urban and 6 rural) (Table 1). These 29 neighborhoods were located within 18 wards (13 urban and 5 rural). The mean age was 46.9 years (SD 15.1). The household non-response rate was 15.0 %. Men (*p* < 0.001) and adults 18–39 years old (*p* = 0.001) were more likely to be non-responders. The median neighborhood cluster size was 13.0 participants (IQR 9–21), and neighborhood cluster size ranged from 6 to 49 participants (Appendix 2).

**Table 1:** Unweighted proportions for demographic, social characteristics, self-reported medical histories, health outcomes, and design parameters stratified by setting; *N* = 481 (CKD-AFRiKA, 2014)

Variable (*n*, %)	Urban	Rural	Total
	(*n* = 370)	(*n* = 111)	(*n* = 481)
Gender (female)	278 (75.1 %)	80 (72.1 %)	358 (74.4 %)
Age			
18–39 years old	138 (37.3 %)	34 (30.6 %)	172 (35.8 %)
40–59 years old	145 (39.2 %)	46 (41.5 %)	191 (39.7 %)
60+ years old	87 (23.5 %)	31 (27.9 %)	118 (24.5 %)
Ethnicity			
Chagga	230 (62.2 %)	58 (52.3 %)	288 (59.9 %)
Pare	35 (9.5 %)	31 (27.9 %)	66 (13.7 %
Sambaa	18 (4.9 %)	9 (8.1 %)	27 (5.6 %)
Other^a^	87 (23.5 %)	13 (11.7 %)	100 (20.8 %)
Education			
None	27 (7.3 %)	4 (3.6 %)	31 (6.4 %)
Primary	253 (68.4 %)	96 (86.5 %)	349 (72.6 %)
Secondary	64 (17.3 %)	10 (9.0 %)	74 (15.4 %)
Post-secondary	26 (7.0 %)	1 (0.9 %)	27 (5.6 %)
Occupation			
Unemployed^b^	71 (19.2 %)	3 (2.7 %)	74 (15.4 %)
Farmer/wage earner	114 (30.8 %)	85 (76.6 %)	199 (41.4 %)
Small business/vendors	143 (38.6 %)	15 (13.5 %)	158 (32.8 %)
Professional^c^	42 (11.4 %)	8 (7.2 %)	50 (10.4 %)
Social characteristics			
Ongoing tobacco use	34 (9.2 %)	16 (14.4 %)	50 (10.4 %)
Ongoing alcohol use	146 (39.5 %)	52 (46.9 %)	198 (41.2 %)
Self-reported medical history			
Diabetes	53 (14.3 %)	8 (7.2 %)	61 (12.7 %)
Hypertension	113 (30.6 %)	21 (19.1 %)	134 (28.0 %)
Stroke	6 (1.6 %)	2 (1.8 %)	8 (1.7 %)
Heart disease^d^	17 (4.6 %)	1 (0.9 %)	18 (3.7 %)
Tuberculosis	10 (2.7 %)	0 (0 %)	10 (2.1 %)
Hepatitis	12 (3.2 %)	2 (1.8 %)	14 (2.9 %)
Malaria	329 (88.9 %)	98 (88.3 %)	427 (88.8 %)
Cancer	6 (1.6 %)	0 (0 %)	6 (1.3 %)
COPD/asthma	23 (6.2 %)	2 (1.8 %)	25 (5.2 %)
HIV/AIDS	20 (5.4 %)	1 (0.9 %)	21 (4.4 %)
Kidney disease	14 (3.8 %)	0 (0 %)	14 (2.9 %)
Health condition			
Hypertension	112 (30.3 %)	37 (33.3 %)	149 (31.0 %)
Obesity	116 (31.4 %)	22 (19.8 %)	138 (28.7 %)
Glucose impairment	102 (27.6 %)	27 (24.3 %)	129 (26.8 %)
Pre-diabetes	63 (17.0 %)	21 (18.9 %)	84 (17.5 %)
Diabetes	39 (10.5 %)	6 (5.4 %)	45 (9.4 %)
Chronic kidney disease	54 (14.6 %)	3 (2.70 %)	57 (11.9 %)
Design parameters			
Neighborhood clusters (*k*)	23	6	29
Median cluster size (IQR)	12.0 (7.5–19.5)	16.0 (15–26)	13.0 (9–21)
Cluster size range	6–49	11–32	6–49
Participants enrolled	370	111	481

^a^Other tribal ethnicities represented in our groups include Luguru, Kilindi, Kurya, Mziguwa, Mnyisanzu, Rangi, Jita, Nyambo, and Kaguru

^b^Included housewives and students

^c^Professional included any salaried position (e.g. nurse, teacher, government employee, etc.) and retired persons

^d^Heart disease included coronary disease, heart failure, or structural diseases

The majority of participants lived in an urban setting (*n* = 370; 77.0 %), were women (*n* = 358; 74.4 %), ethnically Chagga (*n* = 288; 59.9 %), and had obtained a primary school level of education (*n* = 349; 72.6 %), and most participants were occupied as farmers or daily wage-earners (*n* = 199; 41.4 %) (Table 1). Many participants reported an ongoing use of alcohol (*n* = 198; 41.2 %) and many reported a history of malaria (*n* = 427; 88.8 %), diabetes (*n* = 61; 12.7 %), or hypertension (*n* = 134; 28.0 %). Few reported a history of stroke, heart disease, tuberculosis, hepatitis, HIV/AIDS, COPD/asthma, cancer or kidney disease. From our assessment of NCD-related health outcomes, 149 participants (31.0 %) had hypertension, 138 (28.7 %) were obese, 57 (11.9 %) had CKD, and 129 (26.8 %) had glucose impairment of which 84 (17.5 %) had pre-diabetes and 45 (9.4 %) had diabetes.

Clustering varied across neighborhoods and differed by urban or rural setting. Overall ICC coefficients ranged from 0.00 to 0.125 with a mean value of 0.30 (SD 0.033) (Table 2). In the rural setting, ICC coefficients ranged from 0.000 to 0.331, and in the urban setting, ICC coefficients ranged from 0.000 to 0.109. Ongoing alcohol use exhibited the strongest neighborhood clustering (*ρ* = 0.125), which was most prominent in rural neighborhoods (*ρ* = 0.331). Ongoing tobacco use exhibited modest neighborhood clustering in both rural (*ρ* = 0.022) and urban settings (*ρ* = 0.042). Neighborhood clustering of self-reported medical histories was most significant for diabetes (*ρ* = 0.045), hypertension (*ρ* = 0.100), HIV (*ρ* = 0.054), and CKD (*ρ* = 0.020).

**Table 2:** Population-based intra-cluster correlation coefficients (*ρ*) for neighborhood clustering; *N* = 481 (CKD-AFRiKA, 2014)

	Urban		Rural		Overall	
	Mean (SD) or prevalence (%)^b^	*ρ* (95 % CI)	Mean (SD) or prevalence (%)	*ρ* (95 % CI)	Mean (SD) or prevalence (%)	*ρ* (95 % CI)
Social characteristics						
Ongoing tobacco use	15.2 %	0.022 (0.000–0.073)	19.6 %	0.042 (0.000–0.159)	18.0 %	0.028 (0.000–0.075)
Ongoing alcohol use	35.4 %	0.069 (0.000–0.145)	45.8 %	0.331 (0.007–0.654)	41.9 %	0.125 (0.034–0.216)
Self-reported medical history						
Diabetes	9.68 %	0.035 (0.000–0.094)	5.51 %	0.059(0.000–0.194)	7.06 %	0.045 (0.000–0.100)
Hypertension	20.7 %	0.109 (0.012–0.207)	16.1 %	0.014 (0.000–0.101)	17.8 %	0.100 (0.020–0.181)
Stroke	11.8 %	0.000 (0.000–0.039)	2.07 %	0.000 (0.000–0.070)	17.4 %	0.000 (0.000–0.033)
Heart disease	2.64 %	0.000 (0.000–0.039)	0.72 %	0.047 (0.000–0.169)	1.44 %	0.000 (0.000–0.033)
Tuberculosis	2.92 %	0.000 (0.000–0.039)	0.00 %	N/A^a^	1.09 %	0.000 (0.000–0.033)
Hepatitis	2.48 %	0.000 (0.000–0.039)	1.17 %	0.000 (0.000–0.070)	1.66 %	0.000 (0.000–0.033)
Malaria	87.8 %	0.000 (0.000–0.039)	89.0 %	0.001 (0.000–0.073)	88.6 %	0.000 (0.000–0.033)
Cancer	1.04 %	0.009 (0.000–0.039)	0.00 %	N/A^a^	0.39 %	0.000 (0.000–0.033)
COPD/asthma	3.39 %	0.000 (0.000–0.039)	1.31 %	0.000 (0.000–0.071)	2.08 %	0.000 (0.000–0.033)
HIV/AIDS	5.19 %	0.048 (0.000–0.113)	0.59 %	0.010 (0.000–0.093)	0.99 %	0.054 (0.006–0.114)
Kidney disease	3.29 %	0.010 (0.000–0.054)	0.00 %	N/A^a^	1.22 %	0.020 (0.000–0.063)
Health outcome						
Hypertension	19.4 %	0.056 (0.000–0.125)	33.2 %	0.167 (0.000–0.398)	28.0 %	0.075 (0.001–0.126)
Obesity	25.1 %	0.035 (0.000–0.094)	15.6 %	0.009 (0.000–0.091)	19.1 %	0.040 (0.000–0.093)
Glucose impairment	24.0 %	0.025 (0.000–0.078)	20.3 %	0.110 (0.000–0.293)	21.7 %	0.039 (0.000–0.918)
Pre-diabetes	15.8 %	0.001 (0.000–0.040)	16.1 %	0.149 (0.000–0.367)	16.0 %	0.031 (0.000–0.079)
Diabetes	8.21 %	0.000 (0.000–0.039)	4.20 %	0.000 (0.000–0.070)	5.70 %	0.000 (0.000–0.033)
Chronic kidney disease	15.2 %	0.036 (0.000–0.094)	2.03 %	0.000 (0.000–0.070)	7.00 %	0.044 (0.000–0.096)
Physical and laboratory measurements						
SBP (mmHg)	124.0 (24.3)	0.024 (0.000–0.077)	132.8 (26.4)	0.207 (0.000–0.467)	129.5 (24.3)	0.064 (0.000–0.129)
DBP (mmHg)	76.1 (12.3)	0.025 (0.000–0.077)	78.5 (12.1)	0.199 (0.000–0.453)	77.6(12.2)	0.056 (0.000–0.116)
BMI (kg/m^2^)	26.2 (8.23)	0.049 (0.000–0.115)	25.6 (13.7)	0.031 (0.000–0.136)	25.8 (12.0)	0.032 (0.000–0.081)
HbA1C (%)	5.98 (1.28)	0.000 (0.000–0.039)	6.83 (12.7)	0.025 (0.000–0.123)	6.51 (10.1)	0.012 (0.000–0.050)
Serum creatinine (μmol/L)	68.2 (27.2)	0.000 (0.000–0.040)	61.8 (13.6)	0.049 (0.000–0.174)	64.2 (20.0)	0.000 (0.000–0.034)
Albuminuria	14.1 %	0.015 (0.000–0.063)	1.31 %	0.014 (0.000–0.100)	6.08 %	0.038 (0.000–0.090)
eGFR (mg/L/min)(MDRD)	104.7 (24.4)	0.078 (0.000–0.161)	128.5 (203.2)	0.001 (0.000–0.073)	119.7 (161.9)	0.005 (0.000–0.041)
eGFR (mg/L/min) (CKD-EPI)	119.0 (26.6)	0.048 (0.000–0.117)	146.1 (224.3)	0.000 (0.000–0.073)	136.5 (222.1)	0.000 (0.000–0.036)

*SD* standard deviation, *ρ* intra-cluster correlation coefficient, *COPD* chronic obstructive pulmonary disease, *SBP* systolic blood pressure, *DBP* diastolic blood pressure, *BMI* body mass index, *eGFR* estimated glomerular filtration rate, *MDRD* modification of diet in renal disease equation (without the race factor) for eGFR, *CKD-EPI* CKD epidemiology collaboration equation (without the race factor) for eGFR

^a^Too few positive events/outcomes were observed in these categories, ^b^Age-and gender-weighted estimates

**Fig. 1:**
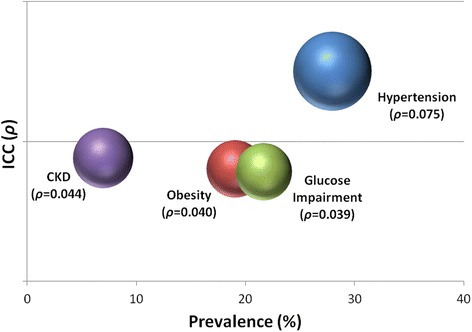
Neighborhood clustering of non-communicable diseases in northern Tanzania. Intra-cluster correlation coefficients, presented by prevalence, for CKD, obesity, glucose impairment, and hypertension

Among the NCDs, neighborhood clustering varied with *ρ* ranging from 0.000 to 0.075. Hypertension (*ρ* = 0.075) exhibited the strongest clustering within neighborhoods followed by CKD (*ρ* = 0.440), obesity (*ρ* = 0.040), and glucose impairment (*ρ* = 0.039) (Fig. 1). Among those with glucose impairment, neighborhood clustering was more significant for pre-diabetes (*ρ* = 0.031) than for diabetes (*ρ* = 0.000). Neighborhood clustering for physical and laboratory measurements paralleled the NCD outcomes. Both systolic (*ρ* = 0.064) and diastolic (*ρ* = 0.056) blood pressures exhibited strong neighborhood clustering. Clustering for albuminuria was modest (*ρ* = 0.038), but it accounted for most of the neighborhood clustering observed for CKD when compared to serum creatinine or eGFR measurements. Similar to obesity and glucose impairment, clustering of BMI was more significant in urban neighborhoods (*ρ* = 0.049) while clustering of HbA1C was more significant in rural neighborhoods (*ρ* = 0.025).

## Discussion

In northern Tanzania, prevalence of NCDs, including hypertension, CKD, obesity, and glucose impairment, exhibited clustering by neighborhood. This clustering varied across urban and rural settings, and for NCD prevalence, it was most significant for hypertension and CKD. Based on the ICC coefficients that we observed, cluster-designed studies examining NCDs in the region should account for the design effect on precision or variance caused by clustering. In a region where the NCD burden is quickly growing, these results will be valuable in designing such studies, including cluster RCTs [5, 12, 23].

The urban and rural differences in neighborhood clustering of NCDs may highlight important environmental and lifestyle risk factors for the development of hypertension, glucose impairment, obesity, and CKD. The neighborhood clustering for hypertension and glucose impairment was most pronounced in the rural settings where families tend to remain more environmentally clustered, share meals, and work in similar agricultural jobs which may all contribute to the development of such NCDs that are known to be highly associated with lifestyle [24–26]. On the other hand, obesity and CKD were most clustered in the urban neighborhoods. For obesity, this urban clustering highlights the importance that urban lifestyles, which may be clustered within neighborhoods on the basis of socioeconomic status, transportation, or occupation, play in the development of obesity. In the context of CKD, living in an urban setting has been shown to be a significant risk factor, yet specific etiologies associated with the urban environment remain unknown [12]. The clustering of CKD within urban neighborhoods that we observed may be important in highlighting causes of CKD, and it further stresses that public health efforts targeting CKD must take a broad approach that includes urban planning with sanitation improvement, safe drinking water, pollution reduction, and infection control.

Among all measured variables, ongoing alcohol use, hypertension, a self-reported history of hypertension, and a self-reported history of HIV were most highly correlated among cluster-sampled individuals, and the latter two variables may reflect an increased awareness and/or prevalence of these conditions within certain neighborhoods. In northern Tanzania, alcohol is commonly homemade and shared among households which may in part explain the significant clustering that we observed.

To our knowledge, this is the first community-based, household-level survey to report on the neighborhood clustering of NCDs in East Africa. As such, these are the first ICC coefficients reported for hypertension, CKD, obesity, and glucose impairment in the region, and compared to reports of ICC coefficients in high-income countries there are significant differences in several of the physical and laboratory variables [27–29]. Because we also measured clustering in both an urban and rural settings we were able to demonstrate important differences which may help inform future studies examining the demographic transition of NCDs in sub-Saharan Africa where rapid urbanization is occurring [30].

Despite these strengths, we also noted a few limitations. Caution must be taken when applying these estimates to other populations and settings. Although the paucity of data currently available for NCD-related measurements and outcomes may make these results useful to researchers more broadly across the region, differences in prevalence and risk factors for NCDs, particularly those that are geographic or environmental-based, mean that even NCDs can cluster at different rates within villages, neighborhoods, or households. Additionally, although we used sample-balancing approaches to address potential non-response bias, the effect of participant non-response upon these estimates is not fully known. Finally, some results, such as self-reported medical history, rely upon the subjective response of individual participants, and as such, they may be prone to recall or response bias.

## Conclusion

In conclusion, we have reported on the observed neighborhood clustering for several NCDs from a community-based study in northern Tanzania. The neighborhood clustering, which varied by urban or rural setting, was substantial enough to contribute to a design effect for NCD outcomes including hypertension, CKD, obesity, and glucose impairment, and it may also highlight NCD risk factors that vary by setting. These results may help inform the design of future community-based studies or randomized controlled trials examining NCDs in the region particularly those that use cluster-sampling methods.

## Appendix 1: Standard Operating Protocol (SOP) for household selection

### Purpose

To provide a reproducible, systematic, and random method of selecting households for sampling.

### Definitions

- Cluster = randomly, pre-selected geographic location that includes multiple households for sampling
- Dwelling = A free-standing building that is covered by a roof. Buildings that share a foundation or appear to share a foundation should be considered as one dwelling.
- Household = Persons residing within a dwelling whose food is prepared by the same person(s)
- ID = Unique Identification Number that is assigned to each participant and each household.
- Household ID = Two digit Unique Identification Number contained in the study ID number that is assigned to each household.
- Eligible Individuals: Adults over the age of 18 who are not pregnant. Ex-pats or Temporary Residents should be excluded unless they are FULL citizens who reside in Tanzania full time (i.e. more than 9 months out of every year).

### Overview

- Cluster site identification
- Household identification
- Household selection process

### Process

1. Cluster site identification: the starting point from which household selection will occur has been identified based on a random GPS coordinates.
2. The dwelling physically closest to the starting point will be approached first.
3. Household Selection Process
  - The first dwelling should be approached:
    i. If that dwelling fulfills the definition of a household then assign it a household ID and assess the eligibility of the household adults according to the enrollment protocol.
    ii. If that dwelling does NOT fulfill the definition of a household then move on to the next dwelling.
    iii. Unless the dwelling is clearly marked as a business, shop, or restaurant then the field surveyors should assume that it could be a household. They should then approach to confirm. (Remember that sometimes people who own shops also live in the back – if any doubt then they should always approach to confirm).
  - To identify the next dwelling to approach for sampling, the following methods should be used:
    i. The field surveyor will stand with his/her back to the main entrance of the first dwelling.
    ii. Flip a coin.
    iii. If the coin lands on TAILS then proceed to your LEFT. If the coin lands on HEADS then proceed to on your RIGHT.
    iv. Next, roll the die to determine which house to approach. The numbers on the die represent which house number (in sequential order according to physical distance to the front door) will be chosen.
    v. If the surveyor comes to an intersection or dead-end before reaching the house number on the die, then flip the coin again to determine the continuing direction. Again, TAILS will be LEFT and HEADS will be RIGHT.
    vi. In instances where there is only one physical direction to go, then proceed in that direction.
    vii. If a dwelling repeats, then repeat the coin-flip and die process.
4. Protocol for Gated Houses
  - House with a Gatekeeper
    i. First, contact the gatekeeper to explain our intentions. If agreeable, he may allow entry.
    ii. If not agreeable to entry, then leave a study overview pamphlet along with our contact information.
    iii. Arrange a follow-up time to see if the owners have expressed interest.
  - Closed Gate House
    i. If a gatekeeper is present then proceed as above.
    ii. If no gatekeeper and no way to contact the household members, then record as non-response.
    iii. Two additional visits, including one off-hours visit (i.e. evening or weekend day), should be attempted according to the follow-up protocol.
  - Open Gate
    i. First, ensure that there is no gatekeeper.
    ii. If no gatekeeper, then approach the household as you would any other dwelling.

## Appendix 2

**Table 3:** Detailed characteristics of the urban neighborhood clusters

Urban neighborhood cluster	1	2	3	4	5	6	7	8	9	10	11	12	13	14	15	16	17	18	19	20	21	22	23	Sum total
Households enrolled (*n*)	15	13	13	7	13	7	24	9	5	18	7	6	6	4	8	5	5	15	43	17	10	9	6	265
Total participants enrolled (*n*)	24	17	24	9	18	9	35	12	8	30	12	6	7	6	11	6	6	21	49	28	14	11	7	370
Participants per household	1.6	1.3	1.8	1.3	1.4	1.3	1.5	1.3	1.6	1.7	1.7	1.0	1.2	1.5	1.4	1.2	1.2	1.4	1.1	1.6	1.4	1.2	1.2	
Gender (*n*)																								
Male	7	2	7	2	4	3	11	4	3	7	2	2	1	2	4	1	2	3	11	7	4	3	0	92
	29 %	12 %	29 %	22 %	22 %	33 %	31 %	33 %	38 %	23 %	17 %	33 %	14 %	33 %	36 %	17 %	33 %	14 %	22 %	25 %	29 %	27 %	0 %	
Female	17	15	17	7	14	6	24	8	5	23	10	4	6	4	7	5	4	18	38	21	10	8	7	278
	71 %	88 %	71 %	78 %	78 %	67 %	69 %	67 %	63 %	77 %	83 %	67 %	86 %	67 %	64 %	83 %	67 %	86 %	78 %	75 %	71 %	73 %	100 %	
Age (*n*)																								
18–39 years old	12	6	6	5	10	1	11	5	3	14	3	2	2	2	3	1	2	9	20	8	7	5	1	138
	50 %	35 %	25 %	56 %	56 %	11 %	31 %	42 %	38 %	47 %	25 %	33 %	29 %	33 %	27 %	17 %	33 %	43 %	41 %	29 %	50 %	45 %	14 %	
40–59 years old	10	7	11	4	4	5	11	3	3	9	6	3	0	0	2	5	4	9	17	15	5	6	6	145
	42 %	41 %	46 %	44 %	22 %	56 %	31 %	25 %	38 %	30 %	50 %	50 %	0 %	0 %	18 %	83 %	67 %	43 %	35 %	54 %	36 %	55 %	86 %	
60+ years old	2	4	7	0	4	3	13	4	2	7	3	1	5	4	6	0	0	3	12	5	2	0	0	87
	8 %	24 %	29 %	0 %	22 %	33 %	37 %	33 %	25 %	23 %	25 %	17 %	71 %	67 %	55 %	0 %	0 %	14 %	24 %	18 %	14 %	0 %	0 %	
Education (*n*, %)																								
None	1	4	3	0	0	0	2	2	0	1	0	0	0	0	0	1	0	3	6	2	1	1	0	27
	4 %	24 %	13 %	0 %	0 %	0 %	6 %	17 %	0 %	3 %	0 %	0 %	0 %	0 %	0 %	17 %	0 %	14 %	12 %	7 %	7 %	9 %	0 %	
Primary	18	9	15	6	15	7	19	9	5	15	8	6	5	3	10	3	5	13	34	21	12	9	6	253
	75 %	53 %	63 %	67 %	83 %	78 %	54 %	75 %	63 %	50 %	67 %	100 %	71 %	50 %	91 %	50 %	83 %	62 %	69 %	75 %	86 %	82 %	86 %	
Secondary	2	3	2	3	2	2	11	0	3	7	4	0	2	3	1	0	0	3	9	4	1	1	1	64
	8 %	18 %	8 %	33 %	11 %	22 %	31 %	0 %	38 %	23 %	33 %	0 %	29 %	50 %	9 %	0 %	0 %	14 %	18 %	14 %	7 %	9 %	14 %	
Post-secondary	3	1	4	0	1	0	3	1	0	7	0	0	0	0	0	2	1	2	0	1	0	0	0	26
	13 %	6 %	17 %	0 %	6 %	0 %	9 %	8 %	0 %	23 %	0 %	0 %	0 %	0 %	0 %	33 %	17 %	10 %	0 %	4 %	0 %	0 %	0 %	
Health outcome (*n*, %)																								
Hypertension	2	7	8	1	2	5	13	2	3	6	3	1	5	4	1	2	0	5	20	13	4	2	3	112
	8 %	41 %	33 %	11 %	11 %	56 %	37 %	17 %	38 %	20 %	25 %	17 %	71 %	67 %	9 %	33 %	0 %	24 %	41 %	46 %	29 %	18 %	43 %	
Obesity	0	7	6	5	4	3	13	2	3	10	3	3	2	2	2	1	2	13	17	8	2	4	4	116
	0 %	41 %	25 %	56 %	22 %	33 %	37 %	17 %	38 %	33 %	25 %	50 %	29 %	33 %	18 %	17 %	33 %	62 %	35 %	29 %	14 %	36 %	57 %	
Diabetes	0	1	2	1	1	2	6	2	0	4	2	0	1	1	0	0	0	1	7	3	2	3	0	39
	0 %	6 %	8 %	11 %	6 %	22 %	17 %	17 %	0 %	13 %	17 %	0 %	14 %	17 %	0 %	0 %	0 %	5 %	14 %	11 %	14 %	27 %	0 %	
Chronic kidney disease	1	4	4	2	4	3	2	5	1	3	0	0	1	0	1	1	1	0	9	9	2	1	0	54
	4 %	24 %	17 %	22 %	22 %	33 %	6 %	42 %	3 %	10 %	0 %	0 %	14 %	0 %	9 %	17 %	17 %	0 %	18 %	32 %	14 %	9 %	0 %

**Table 4:** Detailed characteristics of rural neighborhood clusters

Rural neighborhood cluster	1	2	3	4	5	6	Sum total
Household enrolled (*n*)	22	9	12	14	15	9	81
Total participants enrolled (*n*)	32	11	14	20	18	16	111
Participants per household	1.45	1.22	1.17	1.43	1.20	1.8	
Gender (*n*)							
Male	9	4	5	8	2	3	31
	28 %	36 %	36 %	40 %	11 %	19 %	
Female	23	7	9	12	16	13	80
	72 %	64 %	64 %	60 %	89 %	81 %	
Age (*n*)							
18–39 years old	12	1	2	2	9	8	34
	38 %	9 %	14 %	10 %	50 %	50 %	
40–59 years old	10	5	7	11	8	5	46
	31 %	45 %	50 %	55 %	44 %	31 %	
60+ years old	10	5	5	7	1	3	31
	31 %	45 %	36 %	35 %	6 %	19 %	
Education (*n*, %)							
None	4	0	0	0	0	0	4
	13 %	0 %	0 %	0 %	0 %	0 %	
Primary	27	9	12	16	17	15	96
	84 %	82 %	86 %	80 %	94 %	94 %	
Secondary	1	2	2	3	1	1	10
	3 %	18 %	14 %	15 %	6 %	6 %	
Post-secondary	0	0	0	1	0	0	1
	0 %	0 %	0 %	5 %	0 %	0 %	
Health outcome (*n*, %)							
Hypertension	7	6	6	13	4	1	37
	22 %	55 %	43 %	65 %	22 %	6 %	
Obesity	5	2	5	6	3	1	22
	16 %	18 %	36 %	30 %	17 %	6 %	
Diabetes	2	1	2	0	1	0	6
	6 %	9 %	14 %	0 %	6 %	0 %	
Chronic kidney disease	1	1	0	0	1	0	3
	3 %	9 %	0 %	0 %	6 %	0 %	
